# Potential involvement of miR-375 in the premalignant progression of oral squamous cell carcinoma mediated via transcription factor KLF5

**DOI:** 10.18632/oncotarget.5502

**Published:** 2015-10-12

**Authors:** Wen Shi, Jing Yang, Siyuan Li, Xiaofeng Shan, Xiaosong Liu, Hong Hua, Chuanke Zhao, Zhendong Feng, Zhigang Cai, Lihe Zhang, Demin Zhou

**Affiliations:** ^1^ Department of Oral and Maxillofacial Surgery, Peking University School and Hospital of Stomatology, Beijing, China; ^2^ State Key Laboratory of Natural and Biomimetic Drugs, School of Pharmaceutical Sciences, Peking University, Beijing, China; ^3^ Department of Oral Medicine, Peking University School and Hospital of Stomatology, Beijing, China

**Keywords:** miR-375, OSCC, KLF5, OLP, malignant progression

## Abstract

To elucidate the genetic effect involved in the premalignant progression of chronic inflammation to cancer, we performed microRNA and mRNA profiling in oral lichen planus (OLP), oral squamous cell carcinoma (OSCC), and normal tissue from the same patients. We demonstrate the involvement of a suppressive microRNA, miR-375, in the regulation of this premalignant progression *via* KLF5, a transcription factor that modulates the expression of genes contributing to proliferation and apoptosis. We found that miR-375 abundance decreased in tissues with progression from the normal state to OLP and subsequently to OSCC. Restoration of miR-375 by transduction of a synthetic mimic into OSCC cells repressed cellular proliferation and promoted apoptosis, with concomitant down-regulation of KLF5, and vice versa. The direct binding of miR-375 to the 3′-untranslated region of *KLF5* was further confirmed. Additionally, Survivin (BIRC5), a target of KLF5, was also regulated by miR-375, explaining the susceptibility of miR-375-mimic transfected cells to apoptosis. Further analysis of clinical specimens suggested that expression of KLF5 and BIRC5 is up-regulated during the progression from inflammation to cancer. Our findings provide novel insights into the involvement of microRNAs in progression of inflammation to carcinoma and suggest a potential early-stage biomarker or therapy target for oral carcinoma.

## INTRODUCTION

Oral squamous cell carcinoma (OSCC) is one of the most common types of head and neck cancer. Despite recent advances in therapy, OSCC has a low five-year survival rate, due to late diagnosis and frequent recurrence [1, 2]. Therefore there is an urgent need to elucidate the regulatory mechanisms underlying metastatic progression and identify early-stage molecular signatures that predict tumorigenesis. Epidemiological and retrospective studies suggest that chronic oral mucosa inflammation, such as oral lichen planus (OLP), and human papillomavirus infection are the most prevalent risk factors for OSCC development [3–6]. Chronic inflammation can promote multiple characteristic cancerous processes; the inflammatory microenvironment and inflammatory-induced endogenous oncogenic alterations including microRNAs (miRNAs) or transcriptional changes play decisive roles in tumor initiation and development [7–10]. Recent sequencing and microarray findings have indicated possible genetic effects involved in oral chronic inflammation or OSCC and correlated these with cancer progression. Increasing evidence points to the critical involvement of miRNAs in cancer initiation and progression. For example, Gassling et al. [11] identified disease-associated miRNA-mRNA networks in OLP. Similarly, Cervigne et al. [12] described miRNAs associated with progression of leukoplakia to OSCC based on formalin-fixed paraffin-embedded (FFPE) specimens. However, consistent genetic alterations that contribute to premalignant progression remain largely unknown. The inability to predict molecular signatures may be due in part to the use of unpaired inflammation and carcinoma samples in these studies, since obtaining matching samples from the same patients is difficult. On the other hand, FFPE specimen-based screening provides only miRNA information without the parallel mRNA patterns.

miRNAs are a class of approximately 22-nucleotide non-coding RNAs, which can down-regulate target mRNAs by binding to their 3′-untranslated region (3′-UTR) [13, 14]. miRNAs participate in multiple physiological and pathological processes [15–18]. Several onco-miRNAs, including miR-155, miR-21, miR-196, and miR-210 have been implicated in both inflammation and cancer [19–23]. These miRNAs work as targets of immune and inflammatory stimuli or cancer related transcription factors. By regulating cytokines, transcription factors, or common oncogenic pathways, these miRNAs modulate genomic instability, cellular metabolism, or angiogenesis, which in turn promote malignant progression (reviewed [19]).

In the current study we attempted to quantify global changes in miRNA and mRNA expression in OLP, a typical chronic oral inflammation, and in OSCC from the same patients, in order to identify potential early-stage signatures of oral carcinoma progression. Our results identified a group of aberrantly expressed miRNAs and confirmed that miR-375 is a suppressive miRNA involved in malignant transformation. In addition, we found that miR-375 can bind directly to the 3′-UTR of *KLF5*, encoding an important transcription factor. We provide data showing that miR-375 expression decreases with progression from OLP to OSCC, which may contribute to the over-expression of KLF5. This may promote cellular proliferation as well as decrease cell apoptosis *via* up-regulation of Survivin, resulting in the acceleration of the malignant process.

Concomitant analysis of miRNA and mRNA in such samples is extremely valuable for understanding the genetic contribution to the long-term course of the disease such as the transformation of inflammation into tumors as well as partly eliminating the background noise of individual phenotypes. Moreover, the identification of crucial miRNAs and the related pathways involved in oral malignancy could be beneficial for early-stage diagnosis as well as direct and effective targeted therapy against OSCC.

## RESULTS

### Global miRNA profiling in paired OLP and OSCC tissues reveals the possible involvement of suppressive miRNA, miR-375, in premalignant progression

**Figure 1:**
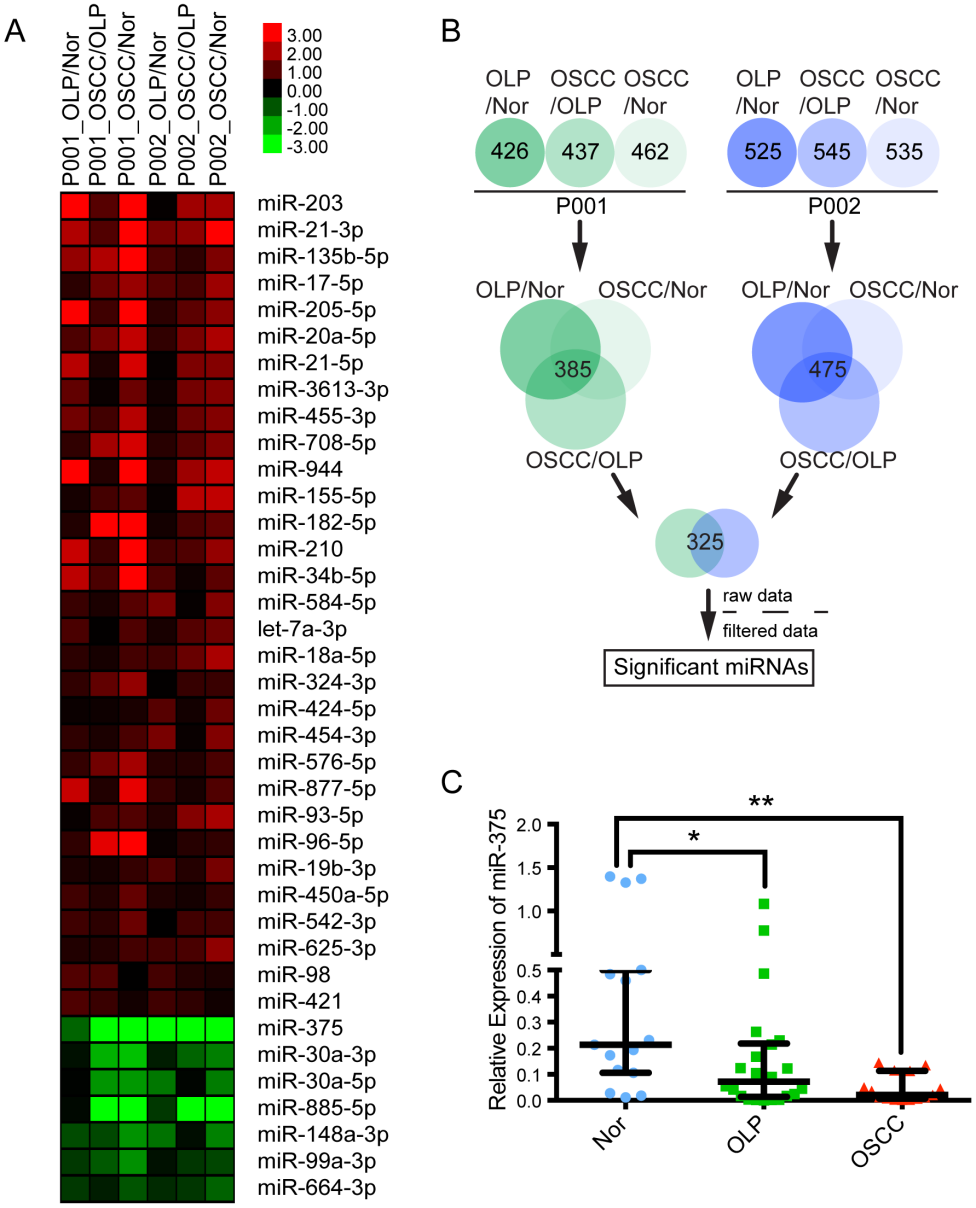
Aberrant miRNAs in OSCC malignant transformation **A.** Expression heat map for the 31 up-regulated and seven down-regulated miRNAs. **B.** Workflow for screening differential miRNAs from NGS data. **C.** miR-375 expression is significantly reduced in OSCC samples compared with adjacent normal mucosa and OLPs (*P < 0.05, **P < 0.01).

To elucidate the genetic effect involved in the premalignant progression of OLP and OSCC, we used next generation sequencing to profile miRNA expression in paired premalignant and tumorous tissues and adjacent normal oral mucosa from the same patients. A comparison of the miRNA profiles of two patients (Supplementary Table 1, Supplementary Figure 1) using a two-fold difference cutoff identified 325 miRNAs differently expressed in OSCC, OLP, and adjacent normal tissues (Figure 1B). Of these, 31 were up-regulated and 7 were down-regulated in all tissues examined (Figure 1A, Supplementary Table 2). miR-375 exhibited high abundance in all tissues but decreased significantly and progressively from normal to OLP to OSCC tissues in both patients, indicating that miR-375 suppression may be involved in the premalignant progress.

To confirm the sequencing results, we examined miR-375 expression in 15 paired OSCC and adjacent normal specimens; miR-375 was significantly down-regulated (*P* < 0.05). Furthermore, the abundance of miR-375 in OLP tissue was lower than in normal tissues (*P* < 0.05), but higher than into OSCC tissue (Figure 1C).

### miR-375 regulates the proliferation and apoptosis of OSCC cells

**Figure 2:**
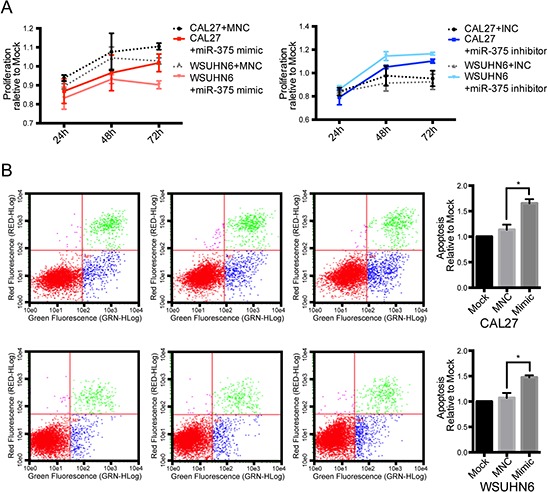
Effect of miR-375 on cell proliferation and apoptosis **A.** treatment with the miR-375 mimic repressed cell proliferation compared with the negative control, while the reverse trend was observed in cells transfected with the miR-375 inhibitor. **B.** the proportion of early apoptosis cells significantly increased in cells transfected with the miR-375 mimic compared with the negative control.

Due to the significant difference in the expression of miR-375 in normal, OLP, and tumor tissue, we sought to determine whether miR-375 plays a key role in the oral malignant process or is merely a downstream result. To examine this question, we introduced a synthetic miR-375 mimic or inhibitor to OSCC cell lines. Our results show that over-expression of miR-375 inhibited the proliferation of CAL-27 and WSUHN6 cells. In contrast, inhibition of miR-375 enhanced cell proliferation (Figure 2A). Furthermore, using flow cytometry to evaluate the effect of miR-375 on apoptosis, we demonstrated that the proportion of early apoptosis cells in both cell lines increased significantly subsequent to transfection with the miR-375 mimic (Figure 2B).

### miR-375 target prediction

The identification of the targets of a miRNA is crucial for understanding its function. Therefore, to identify the potential targets of miR-375 we conducted parallel mRNA profiling and microRNomic analysis in the same samples. Approximately 17744 genes were detected *via* mRNA profiling (Supplementary Table 3). Since miRNAs commonly result in translational inhibition or destabilization of the target mRNA and miR-375 expression was down-regulated in OLP and OSCC tissues, we hypothesized that the targets would most likely be up-regulated genes; using this criterion, 932 up-regulated genes were filtered from our sequencing results (Supplementary Table 4).

**Figure 3:**
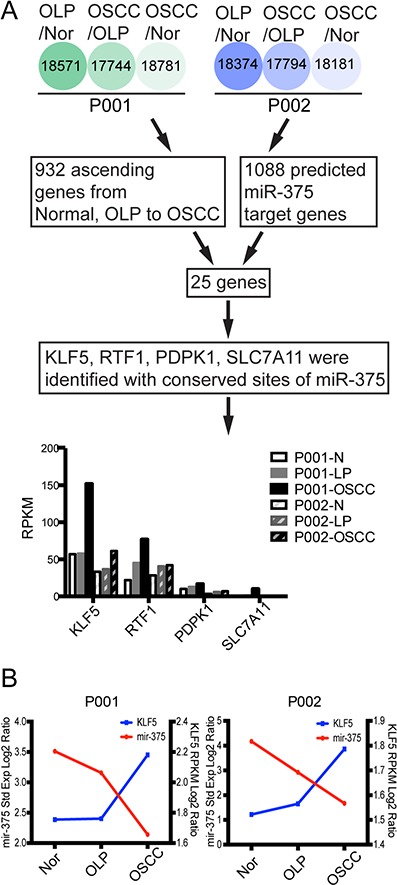
Bioinformatic methods used to predict miR-375 target genes **A.** Workflow for the identification of target genes from NGS data. The expression level patterns of the four predicted genes show that *KLF5* is the predominant target, with the highest expression levels in both patients and a correlating ascending expression pattern. **B.** Co-expression profiles of miR-375 and *KLF5* using mRNA log_2_ ratio RPKM values and miRNA log_2_ ratio standard expression values obtained from the NGS data.

In addition, we utilized miRecords (http://miRecords.umn.edu/miRecords) to predict miR-375 targets and combined these with results obtained using other programs such as TargetScan, miRanda, MiRTarget, PicTar, and RNA22. Using a three program prediction cutoff, we identified 1088 genes as potential targets (Supplementary Table 5). A comparison of the 1088 potential targets with the 932 up-regulated genes, yielded 25 genes (Supplementary Table 6). Next, we used the *TargetScan* database to identify putative targets based on direct binding of the microRNA to the 3′-UTR of genes; four predicted targets were detected: *KLF5*, *RTF1*, *PDPK1*, and *SLC7A11* (Figure 3A).

Transcription factor KLF5 had the highest reads per kilobase transcriptome per million mapped reads (RPKM) values and interestingly, the expression patterns of miR-375 and *KLF5* in normal, premalignant, and cancerous tissues revealed good correlation (Figure 3B).

### Transcription factor KLF5 is an important target of miR-375 in OSCC epithelium cells

**Figure 4:**
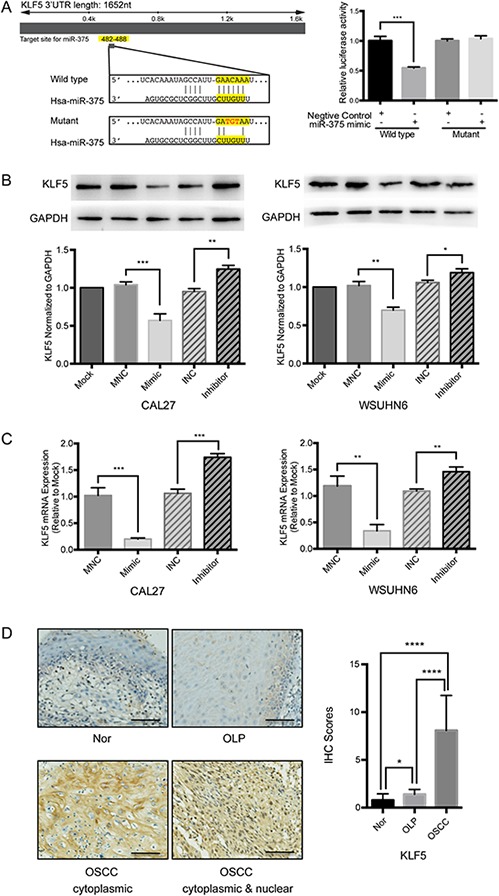
KLF5 is a target gene of miR-375 in OSCC cell lines **A.** Schematic of the wild type and mutated 3′-UTR of *KLF5*. Luciferase activity 24 h post transfection with either the miR-375 mimic or negative control. Luciferase activity of cells co-transfected with the wild type vector and miR-375 mimic was significantly reduced compared with the negative control, while the luciferase activity of the mutant 3′-UTR was not significantly changed. **B.** KLF5 protein levels were significantly repressed 48 h post transfection with 100 nM miR-375 mimic and up-regulated by 200 nM miR-375 inhibitor. **C.** mRNA expression levels were also reduced by the miR-375 mimic and increased by the miR-375 inhibitor 48 h post transfection. GAPDH was used as an internal control. (MNC, miR-375 negative control; INC, mir-375 inhibitor negative control; *P < 0.05; **P < 0.01;***P < 0.001). **D.** The expression pattern of KLF5 was cytoplasmic and mixed cytoplasmic and nuclear. Weak staining of KLF5 was observed in non-tumor and OLP samples, significant differences were observed (magnification, × 400; Scale bar, 25 μm).

miRNAs are known to repress gene expression by targeting the 3′-UTR of mRNA transcripts. In order to determine whether miR-375 regulates *KLF5* directly we constructed vectors containing either the wild type 3′-UTR or mutant 3′-UTR of *KLF5* fused directly downstream of a the firefly luciferase gene (Figure 4A). For the luciferase assays, the wild type or mutant vector was co-transfected into HEK293T cells with either a miR-375 mimic or mimic negative control. The luciferase activity in cells co-transfected with the wild type vector and the miR-375 mimic was significantly reduced compared with the negative control (*P* < 0.001), while the luciferase activity of the mutant 3′-UTR was not significantly altered (Figure 4A). These results strongly suggest that miR-375 binds directly to the 3′-UTR of KLF5.

To evaluate the effect of miR-375 on KLF5 expression we examined both KLF5 protein and mRNA expression levels in miR-375 mimic- or inhibitor-transfected OSCC cell lines. Our results demonstrate that the miR-375 mimic significantly down-regulated KLF5 expression in both CAL27 and WSUHN6, while treatments with the miR-375 inhibitor increased KLF5 levels (Figure 4B, 4C). In addition, immunohistochemistry assays in clinical patient samples indicated that the KLF5 protein was strongly expressed in either or both the cytoplasm and nucleus of OSCC, weakly expressed in OLP, and very weakly expressed in adjacent normal tissue (Figure 4D). These data indicate that miR-375 directly regulates KLF5 expression via a response element within the *KLF5* 3′-UTR.

### Survivin is a putative downstream gene involved in miR-375/KLF5-regulated tumor premalignant progression

In order to identify KLF5 targets that directly participate in miR-375/KLF5-regulated tumor premalignant progression, we conducted an analysis comparing the 932 up-regulated genes with the 50 KLF5 target genes identified using QIAGEN's Ingenuity^®^ Pathway Analysis (IPA^®^, QIAGEN Redwood City, http://www.qiagen.com/ingenuity). Only *KLF5* and *BIRC5*, also known as Survivin, were found in both groups (Supplementary Table 7).

**Figure 5:**
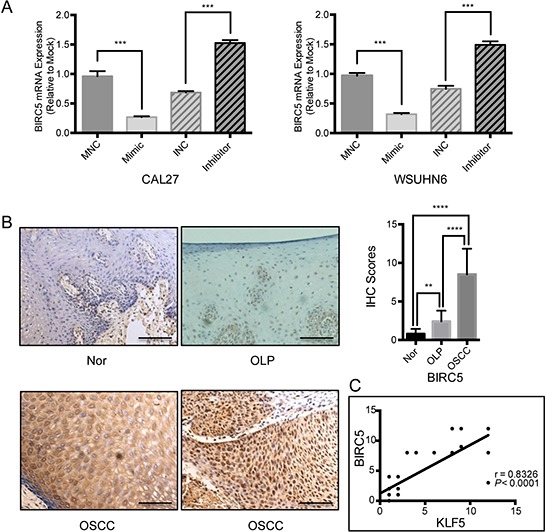
BIRC5 may constitute a downstream gene involved in miR-375/KLF5 regulated tumor premalignant progression **A.** mRNA expression of *BIRC5* was reduced by the miR-375 mimic and increased by the miR-375 inhibitor 48 h post transfection. GAPDH was used as an internal control. **B.** BIRC5 was strongly positively expressed in OSCCs, with a cytoplasmic or mixed cytoplasmic and nuclear pattern. Weak cytoplasmic and no nuclear BIRC5 staining was observed in non-tumor samples, and weakly mixed staining was observed in OLPs (magnification, × 400; Scale bar, 25 μm.). Significant differences were observed among all the different groups (*P < 0.05; **P < 0.01; ***P < 0.001). **C.** the Spearman correlation coefficient between KLF5 and BIRC5 was 0.8326, *P* < 0.0001.

Next, we examined *BIRC5* mRNA expression in miR-375 mimic- or inhibitor-transfected OSCC cells. Treatment with the miR-375 mimic significantly reduced *BIRC5* mRNA levels, while inhibition of miR-375 induced *BIRC5* (Figure 5A). In addition, we examined BIRC5 protein expression by immunohistochemistry using the same clinical specimens used for KLF5 detection. Our results show that BIRC5 expression also gradually decreased from OSCC to OLP to adjacent normal tissues.

Positive signals were located in either the cytoplasm or nucleus or both (Figure 5B). Furthermore, there was a significant correlation between KLF5 and BIRC5 expression; the Spearman correlation coefficient between KLF5 and BIRC5 was 0.8326 (*P* < 0.0001, Figure 5C).

## DISCUSSION

An increasing number of aberrant miRNA expression patterns correlating with pro-inflammatory environments and various cancer types have been reported. In this study, we performed combined next generation sequencing profiling to examine both miRNAs and mRNAs in paired adjacent normal, OLP, and OSCC tissues from the same patients. Our unbiased sequencing data systematically identified 31 progressively up-regulated and 7 progressively down-regulated miRNAs; these constitute interesting candidates for future studies regarding the mechanisms underlying oral epithelium premalignant progression.

Interestingly, the majority of reported inflammation/cancer related miRNAs are oncogenic (up-regulated in cancers). For example, miR-155 and miR-21 have been shown to be up-regulated in a variety of tumors including OSCC, leukemia, colon, lung, pancreatic, and gastric tumors [20–22]; miR-196 is up-regulated in pancreatic and colorectal cancers, esophageal adenocarcinoma, and different types of leukemias [23]; and miR-210 links inflammatory signals with hypoxic microenvironments [24]. Consistent with these findings, we identified three oncogenic miRNAs, miR-21, miR-210, and miR-155, up-regulated in both OLP and OSCC (Figure 1A), providing direct evidence of the involvement of miRNAs in oral chronic inflammation and cancer.

Notably, tumor suppressive miRNA, miR-375, was the most significantly altered in both patients examined, suggesting that suppressive miRNAs contribute to inflammation and cancer progress. This is supported by increasing evidence indicating the causal involvement of suppressive miRNAs in inflammation or cancers [25, 26]. Kozaki et al. have shown that approximately 36.5% of miRNAs are down-regulated in 18 OSCC cell lines [27]. miR-375 was first identified in murine pancreatic cells [28]. Genome-wide miRNA expression profiling studies revealed the extensive presence of miR-375 in various tissues and its significant decrease in cancers, including hepatocellular carcinoma, esophageal carcinoma, gastric cancer, head, and neck cancer [29–33]. Lajer et al. also found that miR-375 was down-regulated in OSCC tissues and suggested that it may function by up-regulating glucose transporters [34]. Characterization of miR-375 in cancers indicates that it acts mainly as a tumor suppressor by repressing several critical oncogene targets [35]. However, few studies have examined the role of miR-375 in the progression from premalignant disease to cancer. In the present study, we determined that miR-375 abundance decreased with progression from the normal state to OLP and subsequently OSCC and that miR-375 can suppress cellular proliferation as well as induce cell apoptosis in OSCC cells, suggesting a suppressive role in malignant transformation.

Since miRNAs function by regulating gene expression at the post-transcriptional level, we hypothesized that miR-375 must down-regulate target genes that promote premalignancy. Based on our parallel transcriptome and microRNomic data, we identified KLF5 as a potential direct target involved in miR-375-regulated premalignant progress. Previous studies have suggested that KLF5 is down-regulated by miR-375 in goblet-cells in mice gut mucosa [36], however, this was not shown directly. To the best of our knowledge, our study is the first to demonstrate the direct binding of human miR-375 to the 3′-UTR of *KLF5*. Furthermore, the miR-375 mimic significantly reduced KLF5 mRNA and protein levels, resulting in improved proliferation in OSCC cells, while inhibition of miR-375 resulted in the opposite effect, suggesting that miR-375 may regulate cell proliferation *via* KLF5 in oral cancer progress.

KLF5, a member of the Kruppel-like family of transcription factors, binds to GC boxes at a number of gene promoters, regulating transcription and the signaling function during cell proliferation, apoptosis, migration, differentiation, and stemness, suggesting that KLF5 functions mainly as a transcriptional activator in human disease including cancers [37–43]. However, recent studies have suggested a contrasting role for KLF5 in carcinogenesis. KLF5 promotes proliferation in esophageal keratinocytes and induces cell death in esophageal cancer cells [44]. KLF5 is generally repressed in prostate cancer samples and cell lines and acts as a tumor suppressor *via* an interaction with ERβ, CBP, or FOXO1 [45–47]. In addition, reduced *KLF5* mRNA levels were demonstrated in breast cancer cell lines [48]. However, consistent with its role as a promoter of proliferation and survival, KLF5 has been implicated as an oncogene in selected epithelial tissues, such as colorectal cancer [49] and intestinal tumors [50]. Here, our *in vitro* results and immunohistochemistry experiments using clinical patient samples both suggest that KLF5 is an oncogene involved in OLP and OSCC.

Although we observed a pro-apoptotic role for miR-375 in our study, the mechanism by which miR-375 contributes to apoptosis is still unknown. Several *in vivo* studies have demonstrated that KLF5 directly inhibits the pro-apoptotic function of PARP1 [51]; overexpression of KLF5 decreased apoptosis in cardiovascular injury associated with reduced cleavage of Caspase-3 [41]. It has also been reported that KLF5 up-regulated anti-apoptotic BIRC5 in human pulmonary vascular smooth muscle cells [52] and acute lymphocytic leukemia cell lines [53]. These results suggest that KLF5 is also a crucial gene mediating the miR-375 pro-apoptotic function. Further analysis of our sequencing data identified BIRC5 as the most likely candidate for the direct effector molecule involved in miR-375/KLF5 apoptosis regulation. Both our *in vitro* and immunohistochemistry results using clinical patient samples are in accordance with this finding.

Immune and inflammatory stimuli such as TNF, IL-1, and IL-6 can regulate the expression of miRNAs. Interestingly, it has been reported that the down-regulation of miR-375 increases sensitivity to human papillomavirus (HPV) infection in exfoliated human cervical cells [54]; HPV infection has been epidemiologically documented as contributing to OLP and OSCC progression [55, 56]. These data suggest that miR-375 may be an important effector molecule in the premalignant progression of oral inflammation to cancer, in response to exogenous stimuli such as HPV infection.

**Figure 6:**
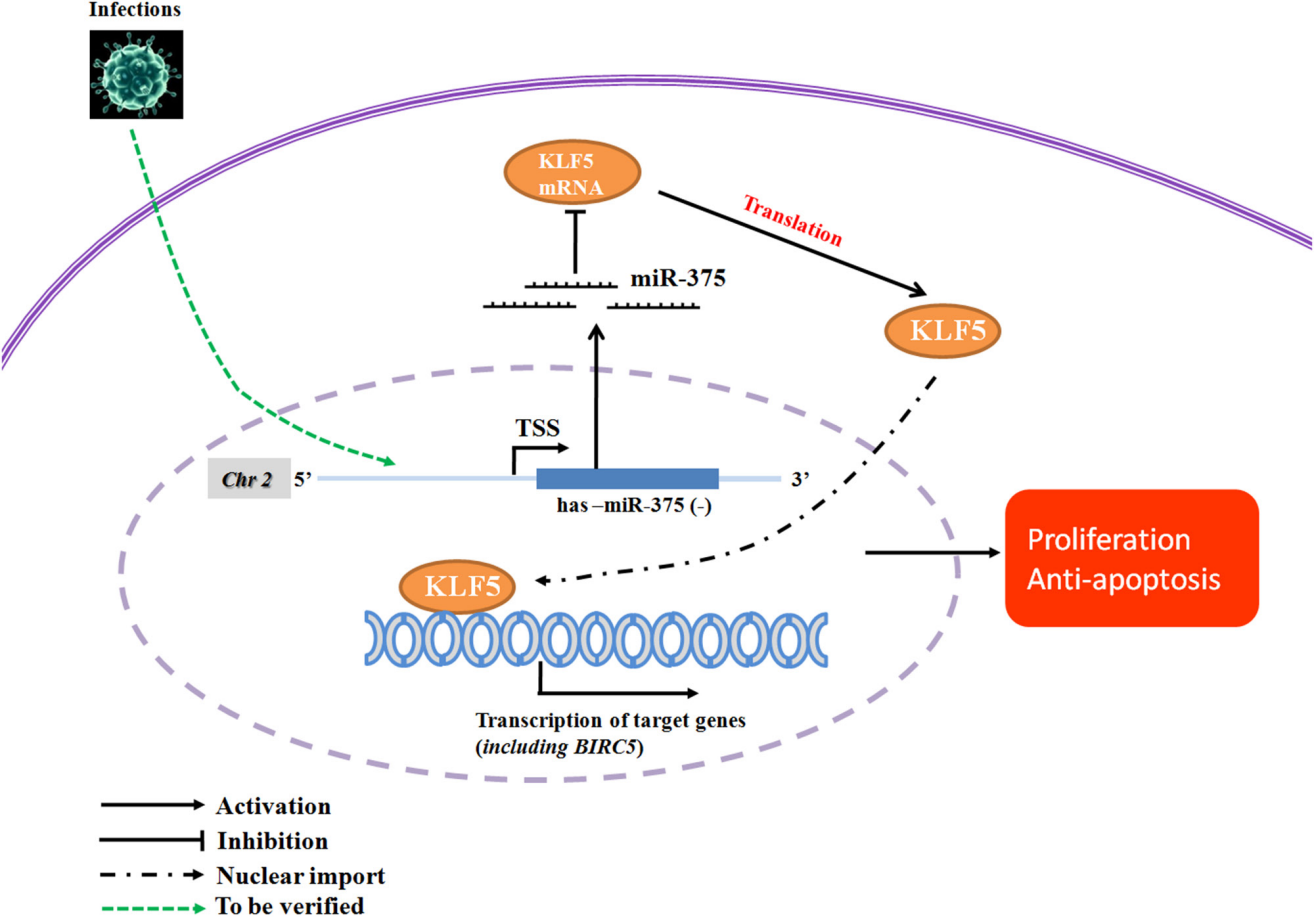
Proposed miR-375/KLF5 regulation mechanism in the premalignant progression of oral squamous cell carcinoma In response to exogenous stimulus such as HPV infection or other inflammation mediators, miR-375 is down-regulated in oral epithelium, which in turn releases the inhibition of KLF5. Up-regulated KLF5 promotes cellular proliferation, as well as decreases cell apoptosis via up-regulation of BIRC5, thus promoting the malignant process.

In conclusion, in this study, paired premalignant and cancer samples from the same patients were used for both mRNA and miRNA sequencing, providing integrated profiles that enable us to identify an association between miR-375, *KLF5* and the downstream gene *BIRC5*. This association might constitute a functional mechanism underlying the malignant progression of OLP and OSCC (Figure 6). These results may provide new options for early diagnosis and treatment of OSCC. However, the early detection feasibility of these targets alone or in combination with other biomarkers needs to be further analyzed in future studies.

## MATERIALS AND METHODS

### Clinical samples

The samples used in this study included paired OSCC, premalignant, and adjacent normal mucosa samples from two patients (P001, P002) and an additional cohort of 15 paired tumor and adjacent non-tumorous mucosa specimens from patients who had undergone OSCC surgery from 2011 to 2014 at Peking University School and Hospital of Stomatology (Beijing, China). The 22 OLP samples were obtained from biopsies. All samples were histologically verified by experienced pathologists. This study was approved by the Peking University Institutional Review Board and all samples were obtained from patients who signed informed consent forms approving the use of their tissues for research purposes following surgery (Approval number IRB00001052–12037). Samples were suspended in RNAlater (Qiagen, Valencia, CA, USA) and stored at −20°C until RNA extraction. Twelve OSCC, nine paired normal mucosa, and 15 OLP paraffin-embedded specimens from operations conducted at the Peking University Hospital of Stomatology from 2008 to 2012 were randomly selected. Medical records and prognostic follow-up data were obtained from the patient database administered by the hospital. The tumors were classified according to the Union for International Cancer Control TNM classification system [57].

### RNA isolation

Total RNA, including miRNA, was isolated from premalignant, tumor, or normal tissue samples using the TRIzol reagent (Invitrogen, Carlsbad, CA, USA) according to the manufacturer's instructions. RNA for next generation sequencing was evaluated with an Agilent 2100 Bioanalyzer (Agilent Technologies, Santa Clara, CA, USA). The RNA integrity number (RIN) for all sequencing samples was between 7.2 and 8.4.

### Next generation sequencing and differential expression analysis

Both mRNA and miRNA were subjected to next generation sequencing (NGS). Quantified total RNA isolated from each sample was used for separate transcriptome sequencing (RNA-seq) and small RNA sequencing (Figure S1A, S1B).

Briefly, for RNA-seq, the mRNA was enriched and fragmented into short fragments (∼200–700 bp). First-strand cDNA was synthesized using random hexamer-primers and the mRNA fragments as templates. Buffer, deoxynucleotides, RNase H, and DNA polymerase I were added for second strand synthesis. The double stranded cDNA was purified with the QIAquick PCR purification kit (Qiagen, Valencia, CA, USA) and then used for end repair and base addition.

Finally, sequencing adapters were ligated to the fragments. The fragments were purified using agarose gel electrophoresis and enriched by PCR amplification. These library products were then sequenced using an Illumina HiSeq^TM^ 2000 (Illumina, Inc. San Diego, CA, USA; Supplementary Figure S1). The primary sequencing data (raw reads) produced by the Illumina HiSeq^TM^ 2000 were subjected to quality control (QC) to determine whether a resequencing step was required. Following QC, the raw reads were filtered, resulting in clean reads which were aligned against the reference sequences using the SOAP2 tool [58]. Only unique mapping tags were used for gene expression analysis. RPKM [59] values were applied to compare gene expression between different samples. The expression fold change for each gene was calculated as the log_2_ ratio of the RPKM values. Subsequently, a strict algorithm was applied to identify differentially expressed genes [60]. The *P*-values for all genes were corrected for multiple tests using a false discovery rate (FDR) adjustment [61].

For miRNA sequencing, the total RNA from each sample was ligated with both a 5′ adapter and 3′ adaptor for reverse transcription, then amplified and purified for sequencing. The small RNAs (sRNAs) obtained from HiSeq deep sequencing covered almost every type of RNA. The sRNAs were annotated by comparing our sequences with those in databases and identifying overlaps in genome location. The experimental process for sRNA sequencing is detailed in Supplementary Figure S1. The same pipeline was used for both mRNA differential expression analysis and miRNA expression analysis.

The NGS data discussed in this publication have been deposited with the NCBI Gene Expression Omnibus [62] and are accessible through GEO Series accession number GSE70666 (http://www.ncbi.nlm.nih.gov/geo/query/acc.cgi?acc=GSE 70666).

### Cell culturing

Two human OSCC cell lines, CAL27 (American Type Culture Collection) and WSU-HN6 (National Institutes of Health) were provided by the Central Laboratory of the School and Hospital of Stomatology, Peking University subsequent to short tandem repeat (STR) identification. The Human Embryonic Kidney 293T (HEK-293T) cell line was obtained from the National Platform of Experimental Cell Resources for SCI-Tech (Beijing, China). All cells lines were maintained in Dulbecco's Modified Eagle's Medium (DMEM; Macgene, Beijing, China) supplemented with 10% fetal bovine serum (FBS; Gibco, NY, USA), 100 U/ml penicillin, and 100 μg/ml streptomycin (Macgene). Cells were cultured at 37°C in a humidified incubator under 5% CO_2_.

### Immunohistochemistry

Briefly, consecutive tissue sections (4 μm) from representative paraffin blocks were deparaffinized in xylene and then rehydrated through graded alcohol solutions. Endogenous peroxidases were blocked using 3% hydrogen peroxide. BIRC5 antigen retrieval was enhanced by microwaving the slides in 0.01 M citrate buffer (pH = 6) for 20 min and KLF5 antigen retrieval was enhanced by microwaving the slides in EDTA (pH 8.0, ZLI-9065_ZLI- 9067, Zhongshan Golden Bridge, Beijing, China). Antibodies against KLF5 (ab24331, Abcam, Cambridge, UK; dilution 1:500) and BIRC5 (ab76424, Abcam, Cambridge, UK, dilution 1:500) were used as primary antibodies. The sections were incubated with the primary antibodies overnight at 4°C then detected using the PV-9000 Polymer Detection System for Immunohistological Staining kit (Zhongshan Golden Bridge). The reaction product was counterstained with hematoxylin. As a negative control, sections were treated with phosphate-buffered saline (PBS) without the primary antibody. Immunostaining for all samples was performed under the same conditions.

A scoring method was used to evaluate KLF5 and BIRC5 expression [63]. The immunostains were reviewed by two independent evaluators. The mean percentage of positive tumor cells was determined by examining 500 cells in at least 5 sections at × 400 magnification. Immunohistochemical (IHC) reactivity was graded according to the percentage of positive tumor cells (a): (0) < 5%, (1) 5–25%, (2) 25–50%, (3) 50–75%, (4) > 75% and the intensity of staining (b): (0) no staining, (1) weak, (2) moderate, and (3) intense staining compared to the negative control. The final evaluation score (c = a × b) was a weighted score calculated for each specimen. The stained tissues were scored blindly in terms of clinical patient data.

### Quantitative real-time PCR (qPCR)

SYBR-based qPCR was used to quantify mature miRNA expression (Quantobio Technology, Beijing, China). *Escherichia coli* DNA polymerase I was used to add polyA tails at the 3′ end of the RNA molecules. Following oligo(dT) annealing, a universal tag was attached to the 3′ end of the cDNAs during synthesis using AMV Reverse Transcriptase (Promega, Madison, WI, USA). qPCR was performed with the tagged cDNA, miRNA-specific forward primers, and a universal reverse primer mix. The qPCR was conducted using the following conditions: 95°C for 5 min followed by 40 cycles of 95°C for 15 sec and 60°C for 60 sec using a Stratagene Mx3005P Real-Time PCR System according to the manufacturer's protocol. The relative microRNA expression level was normalized to that of U6 using the 2^−ΔΔCt^ cycle threshold method [64].

### Oligonucleotide transfection

Synthetic mimics or inhibitors of miR-375 (Ribobio, Guangzhou, China) were transfected into cell line cultures using Lipofectamine 2000 (Invitrogen) to promote or inhibit miR-375 activity, respectively. Negative controls were used for both reactions. The final concentration of the mimics and inhibitors was 100 nM and 200 nM, respectively.

### Cell proliferation assay

The effects of miR-375 expression on CAL27 and WSU-HN6 cell proliferation were assessed using the Cell Counting Kit-8 (CCK-8, Dojindo, Kumamoto, Japan). Briefly, the cells were seeded into 96-well plates. CCK-8 (10 ml) was added to each well at various time points post transfection with either miR-375 mimic or inhibitor, and then incubated at 37°C for 1 h. The absorbance was measured at 450 nm.

### Dual luciferase assay

Both wild type and 3′-UTR-mutated *KLF5* containing the putative seed binding sequence for miR-375 (GAACAAA; nt 482–488) were synthesized and subcloned into the pmiRGLO Dual Luciferase miRNA Target Expression Vector (E1330; Promega) digested with *EcoICR*I and *Xho*I downstream of the 3′UTR of the firefly luciferase used as a primary reporter to monitor mRNA regulation. Renilla luciferase was used as a control reporter for normalization. The reporter constructs were validated by DNA sequencing.

HEK293T cells were seeded in 96-well plates and transfected with 50 nM miR-375 mimic or negative control and 100 ng luciferase reporter plasmid (pmiRGLO-KLF5-WT or pmiRGLO-KLF5-MUT). Luciferase activity was measured 48 h post transfection using the Dual-Luciferase assay kit (Promega) according to the manufacturer's instructions.

### Western blot analysis

Cells were harvested 48 h post transfection and lysed using RIPA lysis buffer containing 1% phenylmethanesulfonylfluoride and 1% protease inhibitor cocktail (Applygene, Beijing, China). Twenty milligrams of protein from each lysate were separated on 12% bis-tris gels (Invitrogen) and transferred to polyvinylidene difluoride membranes. Immunoblotting was performed with diluted (1:500) anti-KLF5 (Abcam) and (1:5000) anti-Survivin (Abcam) antibodies, with the GAPDH antibody (Zhongshan Golden Bridge) serving as an internal control. The membrane was washed and incubated with a goat anti-rabbit IgG (H+L)-HRP conjugate (Zhongshan Golden Bridge) and specific complexes were visualized using Western Chemiluminescent HRP Substrate (Millipore, Billerica, MA, USA).

### Statistical analysis

A non-parametric Mann-Whitney *U* test was used to analyze the relationship between the qRT-PCR numerical values of two groups. For three group qRT-PCR data analysis, a one-way analysis of variance (ANOVA) was used, followed by the Newman-Keuls Multiple Comparison Test for comparing two groups. Paired or unpaired Student's *t* tests were used for tissues and *in vitro* experiments. Linear regression was used to correlate KLF5 and BIRC5 in tissues. Statistical analyses were performed using SPSS 20.0 (SPSS Inc., Chicago, IL, USA) and GraphPad Prism v5.0 software (Graphpad Software Inc, La Jolla, CA, USA).

## SUPPLEMENTARY FIGURE AND TABLES


