# Supplementary material for: Potential involvement of miR-375 in the premalignant progression of oral squamous cell carcinoma mediated via transcription factor KLF5
Source: Oncotarget. 2015 Oct 12;6(37):40172–85. doi: 10.18632/oncotarget.5502 (PMC4741887; doi:10.18632/oncotarget.5502)
Supplement: Supplementary file 1 [file oncotarget-06-40172-s001.pdf]

SUPPLEMENTARY FIGURE AND TABLES

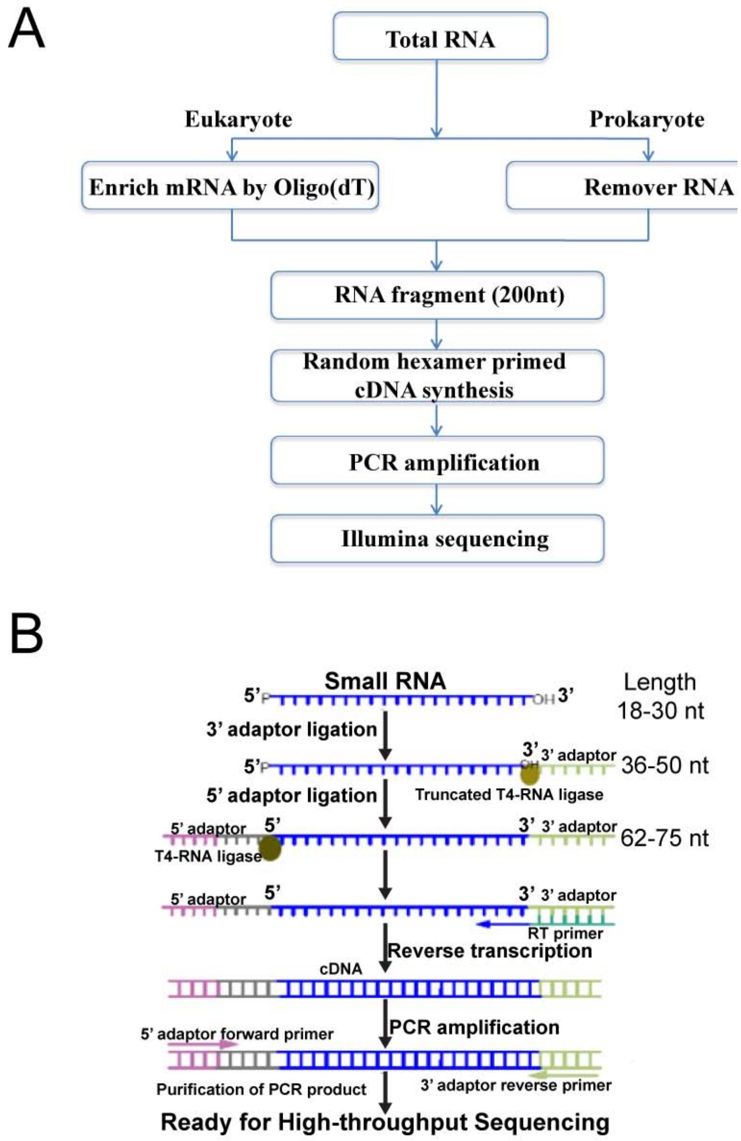

Supplementary Figure S1: RNA sequencing experimental process.

Supplementary Table S1:

Supplementary Table S2:

Supplementary Table S3:

Supplementary Table S4:

Supplementary Table S5:

Supplementary Table S6:

Supplementary Table S7:
